# NURD: an implementation of a new method to estimate isoform expression from non-uniform RNA-seq data

**DOI:** 10.1186/1471-2105-14-220

**Published:** 2013-07-10

**Authors:** Xinyun Ma, Xuegong Zhang

**Affiliations:** 1 MOE Key Laboratory of Bioinformatics, Bioinformatics Division and Center for Synthetic and Systems Biology, TNLIST and Department of Automation, Tsinghua University, Beijing 100084, China; 2 School of Life Sciences, Tsinghua University, Beijing 100084, China

**Keywords:** RNA-seq, Isoform expression estimation, Sequencing bias

## Abstract

**Background:**

RNA-Seq technology has been used widely in transcriptome study, and one of the most important applications is to estimate the expression level of genes and their alternative splicing isoforms. There have been several algorithms published to estimate the expression based on different models. Recently Wu et al. published a method that can accurately estimate isoform level expression by considering position-related sequencing biases using nonparametric models. The method has advantages in handling different read distributions, but there hasn’t been an efficient program to implement this algorithm.

**Results:**

We developed an efficient implementation of the algorithm in the program NURD. It uses a binary interval search algorithm. The program can correct both the global tendency of sequencing bias in the data and local sequencing bias specific to each gene. The correction makes the isoform expression estimation more reliable under various read distributions. And the implementation is computationally efficient in both the memory cost and running time and can be readily scaled up for huge datasets.

**Conclusion:**

NURD is an efficient and reliable tool for estimating the isoform expression level. Given the reads mapping result and gene annotation file, NURD will output the expression estimation result. The package is freely available for academic use at http://bioinfo.au.tsinghua.edu.cn/software/NURD/.

## Background

As the high-throughput sequencing technology develops, using RNA-Seq data to estimate gene expression and isoform expression becomes an important task. There have been some different methods to estimate expression level from RNA-Seq data. Mortazavi et al. used a concept called RPKM to measure the gene expression [1], which had been widely used when alternative splicing is not considered. Jiang & Wong developed a method to estimate the abundance of transcripts of alternative spliced genes [2]. This can be called isoform expression estimation. As it is now known that most human genes can have alternative splicing, estimating isoform expression is becoming a key question in RNA-Seq study. The main idea of Jiang and Wong’s method is to model the sequencing procedure as a random sampling process, and to infer the best estimation of isoform expression by maximizing a likelihood function. However, the method is based on the assumption that reads are uniformly sampled from all transcripts, while many experiments have shown that the distribution of real sequencing reads is not uniform. Instead, read distribution usually has some position-related biases and context-related biases. Based on such observations, people developed some new methods to deal with different types of biases [3-7]. In our experiments, we observed that position-related biases are a major cause of non-uniform distribution and has the most significant influence on expression estimation. We have developed a method to deal with such position-related biases using nonparametric models [3]. The nonparametric nature of the model allows the method to be capable for describing different types of read distributions. Experiments on both simulated data and real data showed that the method can calibrate non-uniform distribution well and outperforms other methods [3]. However, the method was implemented with a set of experimental codes that cannot be scaled up for applications on large RNA-Seq data and this hindered the availability of the method for public use. In this paper, we developed a software NURD as an efficient implementation of the method under C++ using a binary interval search algorithm. The software can handle large-scale data and have shown advantages in both memory use and running time comparing to some other popular software.

## Implementation

### Brief introduction to Wu et al’s method

Wu et al’s method is based on the original method by Jiang & Wong [2]. The original method assumes that the reads are uniformly sampled from the whole transcripts and models the reads count on a specific exon as a Poisson random variable with parameter $\lambda_{j}=l_{j}w\sum_{i=1}^{m}a_{\mathrm{ij}}\theta_{i}$. Here, *m* is the number of isoforms of the specific gene, *θ*_*i*_ is the expression level of the *i*-th isoform, *w* is the total reads count in this sample, *l*_*j*_ is the length of the *j*-th exon and (*a*_*ij*_) is a matrix that indicates the gene structure, with *a*_*ij*_ = 1 or 0 indicating that exon *j* is included or excluded in isoform *i*. Thus the likelihood function of isoform expression based on the observation of *j*-th exon is $L\left(\Theta |x_{j}\right)=\frac{e^{-\lambda_{j}}\lambda_{j}{}^{x_{j}}}{x_{j}!}$. Assuming that exons are independent, we can get the joint log likelihood function of the whole gene as:

(1) $$\begin{array}{l} \log\left(L\left(\Theta |x_{1},x_{2},...,x_{n}\right)\right)=-w\sum_{j=1}^{n}\sum_{i=1}^{m}l_{j}a_{\mathrm{ij}}\theta_{i}\\ \;+\sum_{j=1}^{n}x_{j}\log\left(l_{j}w\sum_{i=1}^{m}a_{\mathrm{ij}}\theta_{i}\right)\\ \;-\sum_{j=1}^{n}\log\left(x_{j}!\right) \end{array}$$

The setting of *a*_*ij*_ = 1 for all exons of an isoform implies that reads are distributed uniformly across the whole isoform. To compensate for non-uniform distribution, Wu et al’s method takes nonparametric models of read distribution across an isoform [3]. It can deal with different kinds of read distribution. The nonparametric read models used include a global bias curve (GBC) for all genes and a local bias curve (LBC) for each gene. The GBC is used to capture the general trend of non-uniform read distribution with regard to the relative position in a gene, shared by all genes in the dataset, and LBC reflects the distribution pattern specific to each gene. Methods for estimating the GBC and LBC curves are described in [3]. Based on GBC curve and LBC curves, we get two corrected gene structure matrices: (GBM)_ij_ and (LBM)_ij_, short for Global Bias Matrix and Local Bias Matrix respectively. The corrected structure matrices are weighted indicator matrices, instead of the 0–1 indicator matrices (*a*_*ij*_). Weights in these two matrices reflect the bias tendency of corresponding exons in this gene. We mix these two matrices as the bias-corrected gene structure which is denoted as (*b*_*ij*_):

(2) $$\left(b_{\mathrm{ij}}\right)=\left(\mathrm{GB}M_{\mathrm{ij}}\right)\alpha +\left(\mathrm{LB}M_{\mathrm{ij}}\right)\left(1-\alpha \right)$$

where *α* is a weight parameter indicating the relative importance of (GBM)_ij_ matrix verse the (LBM)_ij_ matrix in the final gene structure matrix.

By replacing the 0–1 indicator gene structure matrix (*a*_*ij*_) with the weighted gene structure matrix (*b*_*ij*_), we re-define the log-likelihood function as:

(3) $$\begin{array}{l} \log\left(L,\left(\Theta |x_{1},x_{2},...,x_{n}\right)\right)=-w\sum_{j=1}^{n}\sum_{i=1}^{m}l_{j}b_{\mathrm{ij}}\theta_{i}\\ \;+\sum_{j=1}^{n}x_{j}\log\left(l_{j}w\sum_{i=1}^{m}b_{\mathrm{ij}}\theta_{i}\right)\\ \;-\sum_{j=1}^{n}\log\left(x_{j}!\right) \end{array}$$

Further, we can get the gradient of this log-likelihood function by taking derivates for each *θ*_*i*_:

(4) $$\frac{\partial \log\left(L\left(\Theta |x_{1},x_{2},...,x_{n}\right)\right)}{\partial \theta_{i}}=-w\sum_{j=1}^{n}l_{j}b_{\mathrm{ij}}+\sum_{j=1}^{n}\frac{x_{j}b_{\mathrm{ij}}}{\sum_{i=1}^{m}b_{\mathrm{ij}}\theta_{i}}$$

The isoform expression levels can be estimated by maximizing this log-likelihood function. The log-likelihood function has been proved concave in previous work [3] and global optimum can be found by proper optimization algorithm.

### Procedures of NURD

**Figure 1:**
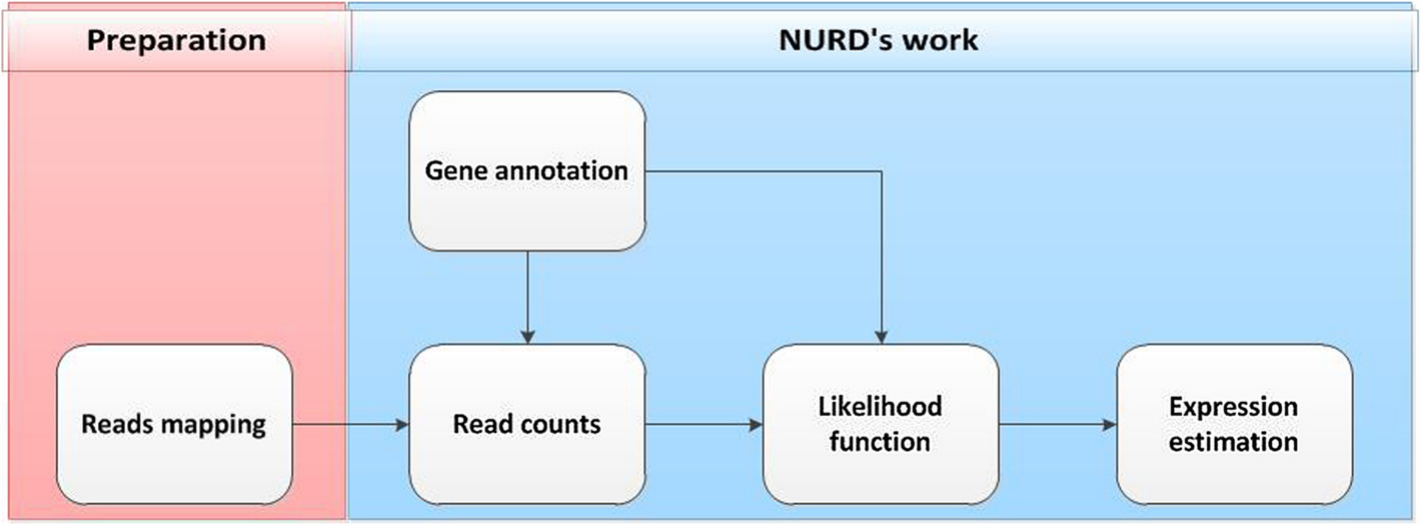
**Flowchart of NURD algorithm.** Using NURD to estimate the isoform expression mainly consists of five steps: **(1)** read mapping, which is not a part of NURD, but the preparation for it; **(2)** getting gene annotation; **(3)** counting reads based on gene annotation and read mapping; **(4)** getting likelihood function based on read counts and gene annotation; **(5)** estimating isoform expression by maximize the likelihood.

The input data of our NURD are the read-mapping file and gene annotation file. The output is isoform expression of each gene. Figure 1 shows the detailed flowchart of the algorithm.

Using NURD to estimate isoform expression typically involves the following five steps.

1 **Read-mapping.** This procedure is not a part of our algorithm, but is a preparation step for it. There have been many read-mapping tools published, such as Bowtie [8] or Tophat [9]. The current implementation of NURD requires that the reads mapping file be in the SAM format.

2 **Gene annotation.** Gene annotation file can be downloaded from a database or assembled by another software such as Cufflinks [5]. The current implementation of NURD requires that the gene annotation file be in the GTF format or refflat format. We extract the basic information of all genes from the gene annotation file, including gene names, number of isoforms and isoform names, number of exons of each gene, length of each exon, and gene structure. Gene structure information tells which exons are contained by each isoform.

3 **Reads counts.** Using reads mapping file and gene annotation file, NURD gets read counts for all exons of all genes in the annotation file.

4 **Log-likelihood function.** Based on the information we get in step 2 and step 3, NURD calculates the GBC of the whole data, LBC for each gene and the bias-corrected gene structure matrix, and furthermore get the corrected log-likelihood function.

5 **Expression estimation.** After the log-likelihood functions are calculated, the major task for NURD is to infer the best expression estimations that maximize the log-likelihood functions. The optimization algorithm we use here is the binary search algorithm, which is very effective and widely used in dealing with search problem [10].

### Implementation of the NURD software

The key step for the efficient implementation of the method is the optimization of the log-likelihood functions for all genes and isoforms. We use the binary interval search technique for the optimization.

### Binary search for single-isoform genes

First, we will illustrate how to use the binary interval search technique to optimize the log-likelihood function if one gene has only one isoform.

The log-likelihood function has been proved to be concave in our previous work [3], so the optimization problem can be transformed to finding the point where the gradient function is equal to zero. Since the objective function is concave and the gene has only one isoform, the corresponding gradient function is a univariate monotone function, in which situation *binary interval search* can be used. Obviously, the log-likelihood function is a real number function and the search space is a real number interval, so the objective of the algorithm is to find a very short interval to cover the optimum point. We initialize the search algorithm with a large enough interval and after each step of binary search algorithm, the length of the interval will shrink to half of the previous interval’s length. As the algorithm goes on, the length of interval will exponentially decrease. As a result, given the precision limit ϵ which is a small real number, the running time complexity of finding the interval covering the optimum point with the gradient equal to zero is O(log(1/ϵ)) .

Gradient ascending algorithm [11] is another technique that is widely used in optimization problem. The binary search algorithm has advantages over the gradient ascending algorithm: Binary search algorithm guarantees to converge to the optimum point in O(log(1/ϵ)), which is really a short time and is fixed given the precision ϵ, while time complexity of gradient ascending algorithm usually depends on step length and the shape of the optimized function. If the step size is not proper, it can be sometime difficult for gradient ascending algorithms to converge. Binary search algorithm doesn’t need to find the proper step size. In some kind of gradient ascending algorithm, there need be another procedure called *line search* for finding a proper step size.

A limitation of binary search compared with gradient ascending algorithm is that the binary search is not as general as the latter for it requires the optimized function to be concave. This is not a problem for the problem in NURD as the log-likelihood function in NURD has been proved concave.

### Coordinate binary search for multi-isoform genes

Because binary search is a 1-dimension search technique, it can only handle the optimization problem of univariate functions. When estimating the expressions of multi-isoform genes, the objective function will be a multivariate function and we need to use coordinate binary search technique. The strategy of coordinate binary algorithm is described in the following pseudo code:

In the innermost loop, we hold all the expressions of isoforms fixed except for some *θ*_*i*_ and the log-likelihood function degenerates to a 1-dimension function. In each step of innermost loop, we maximize the log-likelihood with respect to *θ*_*i*_, given the expressions of other isoforms. Because the log-likelihood function is concave, the 1-dimension objective function is also concave, in which case binary search is suitable.

The pseudo code clearly shows that after each step of the innermost loop, the log-likelihood function will ascend in some degree. As the log-likelihood function is concave, the global optimum will be found after a number of iterations.

**Figure 2:**
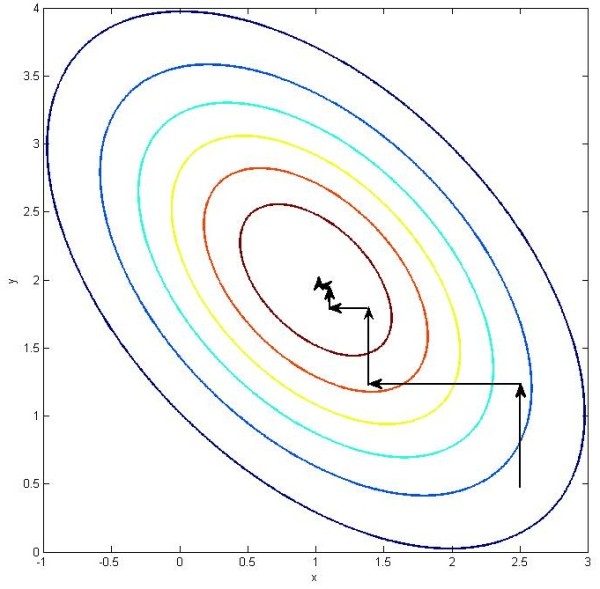
**Illustration of 2-dimension coordinate binary search algorithm.** This is a visualization of a bivariate concave function with its contours. The maximum point is (1, 2). The arrowed line segments show the procedures of coordinate binary search algorithm when optimizing this function. The initial search point is (2.5, 0.5) and search direction is parallel with y axis. In this step of optimization, we fixed x = 2.5 and the bivariate function degenerated in to a univariate function with respect to only y. Since the original function is concave, this degenerated function is also concave and can be optimized by binary search algorithm. After we get the optimum of this degenerated function, the next step of coordinate binary search algorithm is to fix y to the current optimum’s projection on y axis and search in the direction parallel with x axis. These procedures are iterated and the optimum of this function will be finally reached.

The coordinate optimization in 2-dimension case is illustrated in Figure 2.

### Usage of the software

NURD is implemented in C++ and the runtime environment is the Linux systems. The source code is available for free for academic use at http://bioinfo.au.tsinghua.edu.cn/software/NURD/. After getting the source code, one can compile it and get the executable file by simply using *make* command. The software can be efficiently executed with command line inputs. The acceptable gene annotation file format is GTF and refFlat. The acceptable read mapping file is SAM. Short reads should be mapped to genome reference.

The current implementation has not taken the characteristic of paired-end sequencing into consideration, which means NURD regards paired reads as two independent single end reads.

## Results and discussion

In this section, we will mainly focus on the performance of the NURD implementation in terms of accuracy of estimation, computing speed and memory requirement by comparing it with other three published isoform expression estimation related tools. Cufflinks [5] is a widely used tool in RNA-Seq data analysis. It can be used to estimate isoform level expression and assemble the new transcripts based on the RNA-Seq reads. In this manuscript, we will only consider the expression estimation function of Cufflinks for the consideration of fairness of the comparison between different tools. Cufflinks offers an option –G to estimate expression without assembling the transcripts. Cufflinks can also do bias correction if option –b is specified. RSEM [4,6] is another published expression estimation related tool. RSEM is based on a generative model and estimate transcripts’ expression by EM algorithm. When running experiments on RSEM, we will only consider the time and space complexity of parsing reads mapping file and EM algorithm, i.e. the sub-tools named *rsem-parse-alignments* and *rsem-run-em* of RSEM, which are the most important parts of this tool when estimating the isoforms’ expression. Furthermore, we don’t specify the option of *--calc-ci* and no confidence interval is estimated. eXpress [7] is a recently published tool to estimate isoform level expression with RNA-Seq data. It’s based on online EM algorithm, which processes data one fragment at a time.

### Comparing the estimation accuracy on simulated data

A systematic comparison with existing methods on the accuracy of estimating isoform expression has been conducted in Wu et al. [3] that presented the non-parametric method for correcting non-uniform read distribution. Significant advantages over the compared methods have been observed. However, some available tools have been updated since then and some of them also have taken non-uniform distriubion into consideration. Therefore, we further conducted experiments to compare the estimation accuracy of NURD with other recently proposed or updated tools on a set of simulated datasets. The simulations were done with the software flux simulator [12] and the simulated datasets are short read sequences in fastq format. We simulated different sequencing depths, with single-end reads of the length 75 bp sampled from genes of the human genome. Both reference and annotation are from UCSC’s human database (http://hgdownload.cse.ucsc.edu/downloads.html#human). We choose all genes on chromosome 1 to generate the simulated data. NURD and Cufflinks require the reads be mapped to genome reference, while RSEM and eXpress require the reads be mapped to transcriptome reference. Therefore we mapped the reads to both the reference genome and transcriptome.

The measurement of accuracy we use is Major Isoform Recovery Rate (MIRR for short) [3]. MIRR is defined as the percentage of genes whose major isoforms are correctly identified and it’s a robust measurement of the accuracy of some estimated result. Higher MIRR indicates the higher accuracy of estimation. To simplify the comparison, we only focus on the genes on chromosome 1 annotated with two alternative isoforms. We also filter out the genes that share some common exon regions with other genes. There are 391 genes used in total to compare the MIRR of the different tools. The true expression levels in the simulation data can be found in Profile (.PRO) file generated by the flux simulator.

The sequencing depth is defined as the total number of reads generated by software flux simulator. The sequencing depths of our simulating experiments range from 0.01 million reads to 10 million reads sampled from chromosome 1, which covers the typical sequencing depths in current RNA-Seq research.

**Figure 3:**
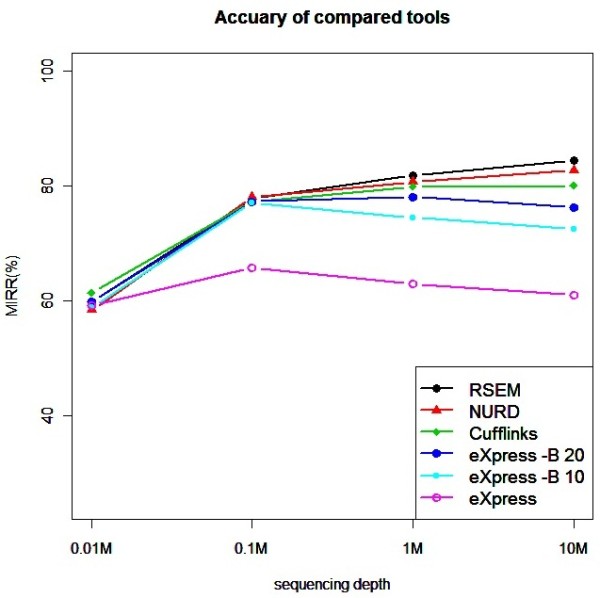
**Comparison of estimation accuracies.** Isoform expression estimation accuracies (measured by MIRR) of compared tools on the simulation data generated with genes on human chromosome 1 with different sequencing depths.

Figure 3 summaries the accuracies of the compared tools on the simulated data. We can see that the accuracy of RSEM, NURD and Cufflinks are very close with each other. All of them perform better when the sequencing is deeper. eXpress does not perform well using the default parameters. By specifying eXpress’s option –B with 10 or 20, which will cause the additional batch EM rounds, the accuracy becomes closer to those of the other methods at moderate sequencing depths, but the performance degrades when the sequencing depth is higher. Also using the –B 10 or 20 option causes the running time to rise to about ten or twenty times of that with the default option (Table 1). Although there’s a significant improvement by specifying the –B 10 option when compared with the accuracy with default option, the further improvement by specifying the –B 20 option is small.

**Table 1:** Comparison of computation performances on 3 typical RNA-Seq datasets

	**Marioni data**		**Yale data**		**Encode data**	
	**(size:3.7 M, length:36 bp)**		**(size:39 M, length:74 bp)**		**(size:213 M, length:76 bp)**	
**Methods**	**Time**	**Memory**	**Time**	**Memory**	**Time**	**Memory**
**NURD**	**36 s**	**60 MB**	**3 m 37 s**	**60 MB**	**19 m 27 s**	**60 MB**
Cufflinks	1 h 31 m 25 s	417 MB	2 h 43 m 17 s	776 MB	3 h 55 m 49 s	2.58GB
RSEM	1 h 38 m 11 s	832 MB	9 h 00 m 55 s	3.65GB	26 h 13 m 6 s	10.3GB
eXpress	17 m 50s	1.44GB	41 m 17 s	1.44GB	1 h 41 m 26 s	1.44GB
eXpress*	1 h 1 m 5 s	1.44GB	3 h 42 m 41 s	1.44GB	13 h 26 m 39 s	1.44GB
eXpress**	1 h 45 m 54 s	1.44GB	6 h 51 m 13 s	1.44GB	25 h 3 m 41 s	1.44GB

eXpress: results with the default parameters.

eXpress*: results by specifying option –B with 10.

eXpress**: results by specifying option –B with 20.

### Comparing running time and memory usage on real data

To compare the running time and memory usage of different tools, we conduct some experiments on three real datasets with different sequencing depths. The first data set is an early RNA-Seq data set published by Marioni et al. [13] [SRA Accession Number: SRA000299]. We refer it as the Marioni data for short. It contains the least and shortest reads among the three datasets. There are about 3.7 M single-end reads of 36 bp length. The second data set is referred as the Yale data [SRA Accession Number: ERP000799], which was submitted by Yale Center for Genomic Analysis, 2013. We call this data as Yale data for short. It represents a moderate scale of current RNA-Seq data. There are about 39 M single-end reads of 74 bp. The largest data set is an ENCODE data with about 213 M paired-end reads of 76 bp [ENCODE Data Coordination Center: wgEncodeEH000140]. This data was granted by Gingeras, CSHL, 2010. We call this data as Encode data for short.

Both Cufflinks and RSEM support multi-threads computation, while NURD does not support in current version of implementation. So we will only consider the single-thread computational mode in our experiments. eXpress will automatically compute in multi-thread mode and the its running time is somehow incomparable with the other three tools. If the computer has only one core, the eXpress’s running time may be longer than the experiments in this manuscript. All the experiments are conducted on an 8-core 2.1GHz linux server with a 32GB RAM.

Table 1 summarizes the running time and memory usage of the compared software on the three datasets. We can see the advantages of NURD over the other three tools on both running time and memory usage are significant. This is partially because that the other three tools are all based on the EM algorithm, which usually requires a number of iterations between E-step and M-step. Each M-step alone needs to solve an optimization problem whose complexity can be comparable with the optimization problem in NURD. Besides, both Cufflinks and eXpress estimate the confidence interval of isoform expression along with point estimation. Cufflinks adopts importance sampling from posterior distribution to do confidence interval estimation, which is usually very time-consuming.

The main reason that NURD consume much less memory than the other three tools is that NURD estimate isoform expression based on the read counts in each exon of each gene. Read counts compress the information of large mapping file into a small space which is only slightly larger than the corresponding annotation information. The computation based on read counts usually can save a lot of running time and consumed memory. The procedure of NURD mainly consists of following three steps: parsing the annotation file, parsing the read-mapping file and expression estimation. The running time and memory usage of the first and last steps roughly scales linearly with the annotation file size, while the time spent on the second step scales linearly only with the number of reads. The memory usage in expression estimation will not increase as the reads number grows, because NURD is based on read counting in exons and the memory usage only scales linearly with the annotation file size. Typically, the total time will increase roughly as a linearly function of the number of reads since RNA-Seq produce more and more short reads and the time spent on reads parsing will dominant the total running time.

## Conclusion

We developed an efficient and robust implementation of Wu et al’s algorithm. It takes the nonparametric read distributions into consideration to improve the accuracy of isoform expression estimation. Experiments on simulated and real datasets have shown that NURD performs one of the best among the compared tools in terms estimation accuracy, and has significant advantage on computational performance. If one wants to get expression estimating from RNA-Seq data both accurately and quickly, NURD could be a competitive alternative.

## Availability and requirements

**Project name:** NURD

**Project home page:**http://bioinfo.au.tsinghua.edu.cn/software/NURD/.

**Operating system(s):** Linux

**Programming language:** C++

**License:** freeware

**Any restrictions to use by non-academics:** None

## Competing interests

The authors declare that they have no competing interests.

## Authors’ contributions

XZ designed the work. XM wrote the software and conducted the experiments. XM and XZ analyzed the results and wrote the manuscript. Both authors read and approved the final manuscript.

## Authors’ information

XM, graduate student of Department of Automation, Tsinghua University, Beijing 100084, China. XZ, Ph.D., Professor of Pattern Recognition and Bioinformatics. Director of Bioinformatics Division, TNLIST. Deputy Director of MOE Key Laboratory of Bioinformatics. Department of Automation, Tsinghua University, Beijing 100084, China.
